# Ear Acupuncture according to the NADA (National Acupuncture Detoxification Association)

**DOI:** 10.3390/medicines6020044

**Published:** 2019-03-31

**Authors:** Gerhard Litscher

**Affiliations:** Research Unit for Complementary and Integrative Laser Medicine, Research Unit of Biomedical Engineering in Anesthesia and Intensive Care Medicine, and TCM Research Center Graz, Medical University of Graz, Auenbruggerplatz 39, EG19, 8036 Graz, Austria; gerhard.litscher@medunigraz.at; Tel.: +43-316-385-83907; Fax: +43-316-385-595-83907

**Keywords:** ear acupuncture, National Acupuncture Detoxification Association (NADA)

## Abstract

This editorial is a brief report on the National Acupuncture Detoxification Association (NADA) ear acupuncture and is intended to briefly summarize the main scientific work. The complementary addiction-detoxification auricular acupuncture method has not been sufficiently experimentally explored in many areas. There have been clinical studies, some of which contradict the success. A total of 27 referenced publications were found that refer to the method that has existed for many decades and should be briefly listed here.

According to the literature, the special ear acupuncture according to the National Acupuncture Detoxification Association (NADA) protocol is an addiction-detoxification method, but in the scientific literature, as in many areas of ear acupuncture, it has not yet been adequately researched in experimental and clinical studies from the point of view of numerous scientific experts. In the scientific database PubMed, as of 21 March 2019, there were a total of 27 referenced publications that refer to the method that has existed for more than 30 years.

Hong Kong’s Dr. H. L. Weng was one of the earliest researchers to report in 1972 and 1973 that acupuncture on four body points and two points on the ear using electrical stimulation was able to support opiate withdrawal in addicts. Cui and coworkers from the Neuroscience Institute of Peking University in China reported on this in both Chinese and English [1,2]. The second step was taken by Dr. Michael Smith of New York, head of NADA/USA in 1985, using only ear points to treat drug abuse. The 1988 publication by Smith and Khan [3] includes reports of 200 patients at the Lincoln Hospital in New York over a period of 13 years, starting in 1975. There have been reports of subjective successes, which, however, were not objectively scientifically proven at that time.

Sixteen years later, ‘NADA acupuncture’ reappears in referenced scientific papers. In Europe, more specifically in Stockholm, the research group around Berman et al. published an article in 2004 [4] using NADA ear acupuncture among prison inmates to alleviate mental and physical complaints and reduce their drug use. Over a period of 18 months, 163 men and women were examined; however, no significant differences to other methods were found, although there were no negative side effects.

The next study also dates back to 2004 and was based on a significant increase in England depending on the use of cocaine among 16- to 29-year-olds. In 2000, England had the highest consumption of cocaine in Europe, affecting 3.3% of all young adults [5]. In a review article, the following question was asked: “Is acupuncture effective in the treatment of cocaine addiction?” Six randomized controlled trials met the inclusion criteria and were considered. The author could not confirm that acupuncture was an effective treatment for cocaine abuse.

Another study on the potential effectiveness of NADA acupuncture in the treatment of cocaine addiction was provided by Kim et al. from Tucson, USA [6]. Again, a positive effect of NADA acupuncture could not be clearly demonstrated. The lack of clinical trials is explicitly stated, and the authors state that further efforts should be made in this area [6].

The next international study in a peer-reviewed journal of NADA acupuncture was from Margolin et al. from the Yale University School of Medicine, New Haven, USA [7]. In this 2005 study, 40 HIV-positive cocaine users received acupuncture according to the NADA protocol (Figure 1). In addition to the acupuncture treatment, patients also received group therapy. Depression and anxiety were examined. The results showed no measurable success of acupuncture. However, patients who received group therapy in addition to acupuncture treatment were formerly abstinent and showed a greater reduction in depression and anxiety than those who did not receive group therapy.

In another study performed in 2011 in Canada, subjects were assigned to one of three treatment groups (NADA auricular acupuncture, auricular acupuncture at sham points, or treatment setting control). Anxiety was assessed using a pretest–posttest treatment design. In this study, the NADA protocol was not more effective than the sham or treatment setting control in reducing anxiety [8]. NADA was also used at Danish rehabilitation institutions and drug centers [9]. Motives for offering NADA acupuncture were most often some positive experienced effects. A randomized controlled trial design with the objectives to evaluate the feasibility and possible benefits of self-administered auricular acupressure (NADA) as a non-invasive alternative to pharmacotherapy has also been suggested for smoking cessation [10]. Interviews in Sweden were conducted with 15 patients treated at an outpatient clinic for substance dependence [11]. All participants appreciated NADA treatment. “Auricular acupuncture for chemically dependent pregnant women: a randomized controlled trial of the NADA protocol” is the title of an interesting study, again from Canada [12]. The authors observed that among the newborns of women who were compliant with the acupuncture regime, there was a reduction of 2.1 and 1.5 days in the length of treatment for neonatal abstinence syndrome when compared to the non-compliant and control groups, respectively. However, the differences were not statistically significant. Another article from New York describes the auricular acupuncture and acupressure program developed for a university setting and its use as a tool to enhance harm reduction and mental health services [13]. In a publication from Beijing, the efficacy of acupuncture and related techniques for the treatment of drug dependence was reviewed. Possible mechanisms underlying these effects were also discussed [14]. At the University of Colorado in Denver, 185 patients completed a study. The use of NADA acupuncture was positively correlated with both the successful completion of the study as well as successful tobacco cessation [15]. Other authors from the USA reported that NADA acupuncture is a simple, standardized, 1–5 point auricular needling protocol that is increasingly recognized as a universally useful intervention in the treatment of addictions [16]. Researchers from the Medical University of Graz in Austria (TCM Research Center Graz) used NADA ear acupuncture in an adolescent patient with phantom limb pain after surgery for osteosarcoma [17] and authors from Germany implemented the method in geriatric patients suffering from major depression [18,19]. Significant pre–post improvements indicated a potential benefit [18]. Impulsivity has characteristics that are manifested clinically in behaviors such as disinhibition, poor self-control, lack of deliberation, thrill seeking, and risk-taking. NADA acupuncture also holds promise as a useful treatment adjunct in the management of disorders for which impulsivity is a prominent component [20]. On the one hand, the results from a publication in Neuroscience Letters from 2016 provides some support for the evidence-based use of NADA acupuncture as a new adjunctive approach that can improve the side-effects of morphine and other opioids [21]. On the other hand, no evidence was found that NADA acupuncture was more effective than relaxation for problems with anxiety, sleep or substance use, or in reducing the need for further addiction treatment in patients with substance use problems and comorbid psychiatric disorders [22]. Results from Germany showed that treatment with NADA acupuncture in patients with anxiety disorders or major depressive disorders significantly decreased tension, anxiety, and anger/aggression, but did not elevate mood. Between NADA acupuncture and progressive muscle relaxation, no statistically significant differences were found. Thus, the authors suggest that both methods may be useful as equally-effective additional interventions in the treatment of the above mentioned disorders [23]. 

Stuyt and Voyles stated that NADA acupuncture has evolved into the most widely implemented acupuncture-assisted protocol, not only for substance abuse, but also for broad behavioral health applications [24]. Promising, early randomized-controlled trials were followed by a mixed field of positive and negative studies. The authors recommended that, going forward, research continues to explore the comparison of the NADA protocol added to accepted treatments to those treatments alone, recognizing that it is not a stand-alone procedure, but a psychosocial intervention that affects the whole person and can augment outcomes from other treatment modalities [24].

“NADA Protocol for Behavioral Health. Putting Tools in the Hands of Behavioral Health Providers: The Case for Auricular Detoxification Specialists” is the title of an article published in Medicines recently in 2018 [25]. Data presented there support the idea that conditions conducive to auricular detoxification specialist practice led to greater implementation [25].

Baker and Chang [26] from the USA performed a systematic review at the end of 2016, which was the first to focus explicitly on randomized trials utilizing the NADA protocol as a complementary intervention to address opioid use disorder. Only four trials met the inclusion criteria. The results indicated that while the NADA protocol may not be effective in reducing acute opiate craving or withdrawal, it may be effectively utilized as an adjunctive treatment to increase treatment retention and decrease methadone detoxification and maintenance dosages in opioid use disorder [26]. In a randomized prospective study performed in 2017 to determine if NADA plus traditional treatment enhanced outcomes, participation in NADA showed better positive results [27].

## Figures and Tables

**Figure 1:**
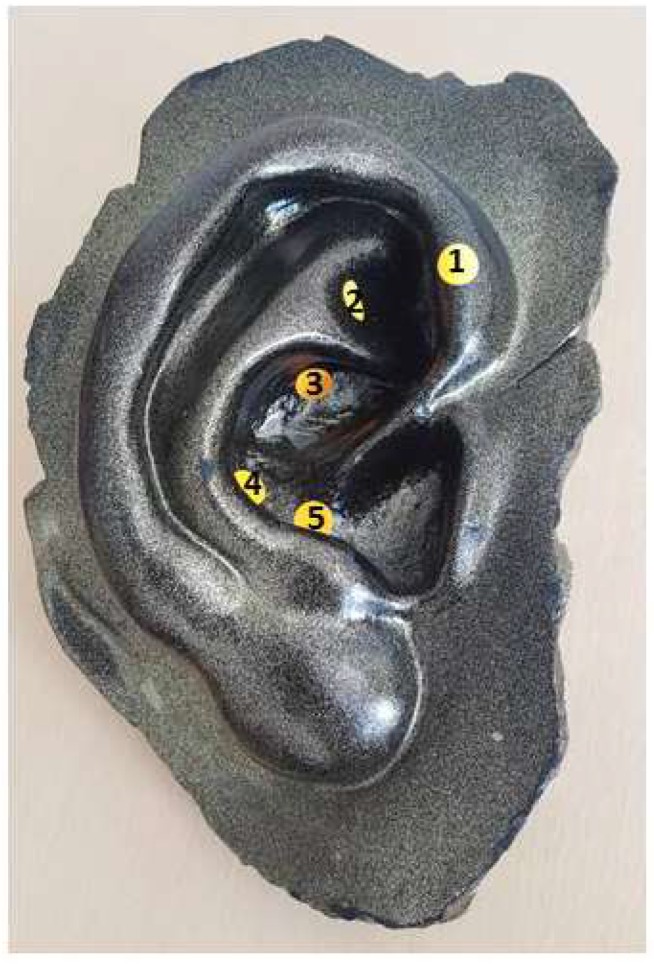
NADA auricular points (schematically): (1) Sympathetic, (2) Shen Men, (3) Kidney, (4) Liver, (5) Lung.
